# Impact of snow-darkening by deposition of light-absorbing aerosols on snow cover in the Himalaya-Tibetan-Plateau and influence on the Asian Summer monsoon: A possible mechanism for the Blanford Hypothesis

**DOI:** 10.3390/atmos9110438

**Published:** 2018-11-12

**Authors:** William K. M. Lau, Kyu-Myong Kim

**Affiliations:** 1 Earth System Science Interdisciplinary Center, U. of Maryland; 2 Climate and Radiation Laboratory, NASA/Goddard Space Flight Center

**Keywords:** snow-darkening, light-absorbing aerosols, dust and black carbon, elevated-heat- pump effect, snow cover-monsoon relationship, Blanford hypothesis

## Abstract

The impact of snow darkening by deposition of light absorbing aerosols (LAAs) on snow cover over the Himalaya-Tibetan-Plateau (HTP) and influence on the Asian summer monsoon are investigated using the NASA Goddard Earth Observing System Model Version 5 (GEOS-5). We find that during April-May-June, deposition of LAAs on snow leads to a reduction in surface albedo, initiating a sequence of feedback processes, starting with increased net surface solar radiation, rapid snowmelt in HTP and warming of the surface and upper troposphere, followed by enhanced low-level southwesterlies and increased dust loading over the Himalayas-Indo-Gangetic Plain. The warming is amplified by increased dust aerosol heating, and subsequently amplified by latent heating from enhanced precipitation over the Himalaya foothills and northern India, via the Elevated Heat Pump (EHP) effect during June-July-August. The reduced snow cover in the HTP anchors the enhanced heating over the Tibetan Plateau and its southern slopes, in conjunction with an enhancement of the Tibetan Anticyclone, and the development of an anomalous Rossby wavetrain over East Asia, leading to weakening of the subtropical westerly jet, and northward displacement and intensification of the *Mei-Yu* rainbelt. Our results suggest that atmosphere-land heating induced by LAAs, particularly desert dust play a fundamental role in physical processes underpinning the snow-monsoon relationship proposed by Blanford more than a century ago.

## 1. Introduction

Since Blanford [1] first reported a possible inverse relationship, *i.e.,* increased Himalayan snow cover linked to a weakened South Asian summer monsoon (SASM), nearly a century had elapsed before researchers began the rigorous pursue to better understand the relationship using modern satellite observations and global climate models. Modern studies of snow-monsoon relationship have provided more diverse perspectives of the original Blanford hypothesis that can be broadly categorized into three overlapping strands. Strand-1 represents work done mostly in the 1970–1990’s, in which the original Blanford hypothesis was essentially affirmed, but with additional findings that the relationship might be a component of a broader connection between Eurasian boreal winter and spring snow cover and the South Asian Summer Monsoon (SASM) [2–10]. In Strand-2 (1980 to mid-2000’s) thanks to the advances of modern satellite data, global reanalysis data and global climate models, studies of Eurasian snow cover-monsoon relationships were expanded to include possible impacts on East Asian summer monsoon (EASM), and connections with major modes of global climate variability. Evidence were found that the snow-monsoon relationship could be attributed to the influence of El Nino Southern Oscillation (ENSO) on both snow cover and monsoon, and that the relationship is strongly masked by the influence of ENSO [11–17]. Included in this strand were also studies showing that the different patterns of Eurasian snow cover were controlled by various modes of natural climate variability, and that during certain periods, increased SASM precipitation was found to be preceded by above normal winter and spring snow cover over the Tibetan Plateau, contrary to the Blanford hypothesis [18–19]. Others have surmised that that a weakening of the snow-monsoon relationship in recent decades may be related to a weakened ENSO-monsoon relationship, possibly due to climate change [20–21].

Strand-3 represents more recent studies from the mid-2000’s to the present, where the focus is back on the fundamental physical underpinnings of the Blanford’s snow-monsoon relationship, by removing or minimizing impacts of remote SST forcing from climate variability. Corti et al [22] found strong inverse relationship between Himalaya winter snow cover and Indian monsoon, unless a strong ENSO is present. Wu and Kirkman [23] noted that while ENSO and Tibetan snow cover compete for influence on the Indian monsoon, they cooperate to enhance monsoon precipitation over southern China, via a wave train signal connecting the two regions. Fasullo [24] used a stratified diagnostic methodology and found that in ENSO neutral years, the inverse relationship between Himalayas-Tibetan Plateau (HTP) snow cover and Indian monsoon rainfall was highly significant, while for Eurasian snow cover the correlation with Indian monsoon rainfall is only modest. Turner and Slingo [25] found similar results in numerical model experiments using the HadCM3 coupled model, indicating that an increase in surface albedo due to more snow cover over the HTP is key to reduction in surface fluxes leading to a cooling of the Tibetan Plateau, and reduced meridional tropospheric temperature gradient during the early summer, and therefore a weaker SASM. In related modeling studies, Wang et al. [26] found that a warmer and less snow-covered Tibetan Plateau (TP) could lead to increased summer monsoon precipitation over northern India, and an enhanced subtropical *Mei-Yu* rain belt over East Asia, in conjunction with the development of an upper-troposphere Rossby wave train, spanning the TP and subtropical East Asia. Overall, in all three strands, studies were largely based on observational correlative analyses, which were highly dependent on the spatial and temporal windows chosen. So far modeling studies to isolate the snow-monsoon relationship have been limited to using various prescribed idealized snow cover changes as boundary forcing to the atmosphere, without consideration of the feedback processes involving snowmelt and atmospheric dynamics. There is a relative dearth of process-oriented modeling studies, especially with regard to the physics of snowmelt. As a result, the fundamental physical processes involved in snow cover change, and interactions with monsoon dynamics remain poorly understood.

Contemporaneous with the Strand-3 studies, there has been an explosive growth in studies of the aerosol-monsoon interactions, indicating that ambient aerosols, both natural and anthropogenic, through direct (radiative) and indirect (microphysical) effects could have a strong impact on Asian monsoon forcing, variability and change [27–39]. Shielding of solar radiation by aerosols, *i.e.,* solar dimming effect, cools the land surface over Asia, reduces land-sea contrast and thus weakens the monsoon [28]. On the other hand, atmospheric heating by light absorbing aerosols (LAAs), *i.e.,* black carbon (BC), organic carbon (OC) and desert dust over the Himalayan foothills and Indo-Gangetic Plain can heat the atmosphere, and induce diabatic heating and dynamical feedback via the so-called Elevated Heat Pump (EHP) effect, that could strengthen the early Indian summer monsoon, accelerate the melting of HTP snowpack, as well as modulate ENSO influence on the South Asian monsoon [30, 32, 41–47]. On seasonal and intra-seasonal time scales, effect of absorbing aerosols may affect the timing and duration of monsoon active and break periods, as well as advance the monsoon rainy season with increased frequency of extreme precipitation over the Himalayas foothills and northeastern India [48–50].

Another important impacts of aerosol on climate stems from the reduction of surface albedo by deposition of LAAs on snow surface, i.e., the snow darkening effect (SDE), causing increased absorption of surface solar radiation and warming of the extratropical land surface, and high mountain regions. During boreal spring, over the snow-cover regions of Eurasia, SDE far exceeds the aerosol dimming effect, resulting in strong positive radiative forcing [51–52]. The efficacy of snowmelt over the HTP, defined as the amount of snow-cover reduction per unit rise in surface warming is much larger due to LAAs than greenhouse warming [53]. Equilibrium climate model experiments show that SDE warms the extratropical Eurasian land surface by up to 2° C, compared no-SDE experiments, exerting significant impacts on the water and energy balances and hydro-climate of the Northern Hemisphere continents [54–57]. Based on historical data, significant quantities of LAAs have been found in snowpack and glacier in the HTP due to local emissions over South Asia, as well as remote transport from afar, causing increases surface solar radiation absorption via SDE by 5–20 Wm^−2^ [54, 58–59]. However, the roles of SDE on HTP snow cover on the Asian summer monsoon, and relevance to the snow-monsoon relationships have not been investigated. The objective of this paper is to shed new light on physical processes involving snowmelt induced by SDE over the HTP, and subsequent interactions with aerosol transport, atmosphere-land heating processes, and monsoon dynamics.

## 2. Model and Methodology

To tease out the SDE impacts on the Asian summer monsoon (ASM), we have carried out numerical experiments using the NASA Goddard Earth Observing System Model Version 5 (GEOS-5) climate model [60], under prescribed sea surface temperature (SST) and polar land-ice and sea-ice conditions. The land surface model in GEOS-5 is the catchment model [61–62], which uses the snowpack model of Lynch-Stieglitz [63]. Here, we use the newly developed GOddard SnoW Impurity Model (GOSWIM) snow darkening physics package which includes radiative transfer calculations of snow albedo and mass distributions of deposited constituent aerosols of dust, BC, and OC in snow [64–65]. Aerosol emission, transport and radiative processes are provided by the Goddard Chemistry Aerosol Radiation and Transport (GOCART) module [66]. The LAAs in GOCART consist of wind-generated mineral desert dusts [67], prescribed climatological black carbon (BC) and organic carbon (OC) emissions from anthropogenic and natural sources including fossil fuel and biomass burning [68–69]. The version of GEOS-5 used in this study does not include effects of aerosol-cloud microphysics interactions [70].

Two sets of 10-member ensemble experiments have been carried out. Each member consists of a 10-years simulation forced by prescribed observed SST from 2002–2011 [71], but with different atmospheric initial conditions, using the GEOS5 model at 2° x 2.5° latitude-longitude horizontal resolution and with 72 vertical layers. The first set of experiments (referred to as SDE) employs the fully interactive land surface and snow processes including the GOSWIM SDE physics module. The second set of experiments (referred to as NSDE) is identical to the first except for the absence of SDE physics, *i.e.,* constituents are not tracked in the snowpack, and do not affect the snow’s surface albedo. Atmospheric heating by LAA’s are included in both SDE and NSDE. The impact of SDE on monsoon climate are evaluated based on anomaly fields, defined as the difference in the ensemble mean climatology of the two experiments (SDE - NSDE), each climatology being based on the full 100 years of simulation (10 years x 10 ensemble members). Statistical significance of the results is evaluated using the Student’s t-test. Further details of the model setup and comparison of ensemble mean climates of SDE and NSDE can be found in two related previous studies based on the same experiments, revealing the importance of SDE on continental scale water and energy balances over northern hemisphere continents [56], as well as on hydro-climate feedback, increasing frequency of heat waves over extratropical Eurasia land, during boreal spring and summer [57]. Model climatologies of monsoon rainfall, winds, temperature, aerosol optical depth (AOD), snow cover are validated with data from the Tropical Rainfall Measuring Mission (TRMM), the NASA Modern Era Retrospective-analysis for Research and Application, Version-2 (MERRA2) reanalysis, and MODerate-resolution Imaging Spectroradiometer (MODIS) respectively. In this work, we focus on the physical mechanisms of SDE impact on snow cover in the Himalayas-Tibetan Plateau (HTP) and subsequent influence on the ASM.

## 3. Results

In this section, we discuss in order, a) the monsoon-snow-aerosol climatology and comparison with observations, b) SDE-induced forcing and dynamical feedback processes, c) changes in the mean monsoon equilibrium climatic states, and d) implication of our results on the fundamental physical underpinning of the Blanford hypothesis. At the outset, it is important to point out that the model LAAs considered here consist of only primary aerosols, *i.e.,* desert dust, BC and OC. Chemical processes and secondary aerosol formation are not included. Dust aerosols, as in nature, are treated as intrinsic component of the aerosol-snow-monsoon climate system, with emission rates internally generated as functions of surface winds, atmospheric stability, and soil conditions over deserts and semi-arid regions. On the other hand, model BC and OC emissions are prescribed with seasonal climatology, including both natural and anthropogenic sources. All aerosols are transported by winds and subject to removal by both dry and wet depositions. In this work, we focus on the interactions of monsoon dynamics with ambient total aerosols (anthropogenic + natural) on sub-seasonal to seasonal time scales. For brevity, we refer to the sum total of BC and OC as carbonaceous aerosols (CA) in the following discussion.

### 3.1 Monsoon-snow-aerosol climatology

**Figure 1.**
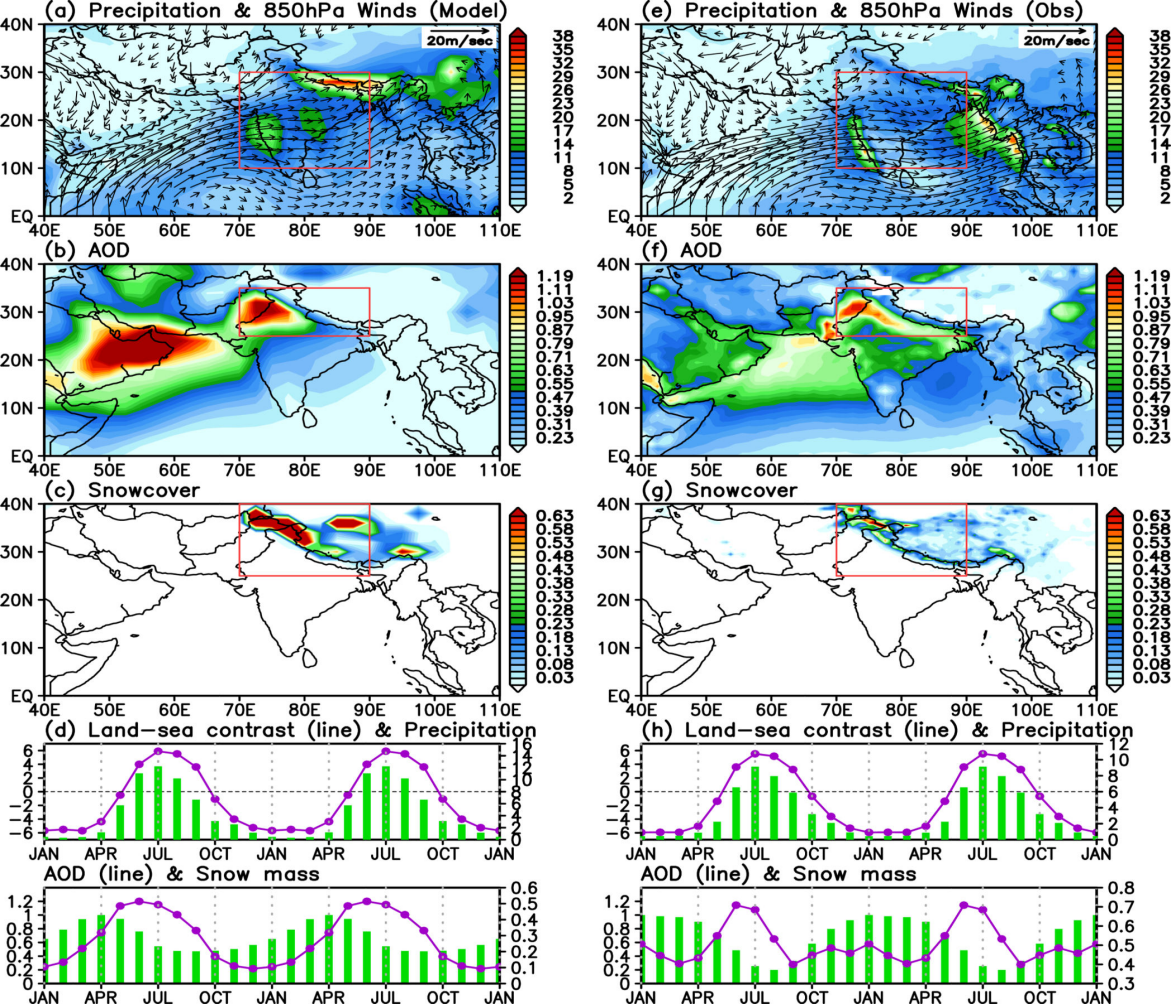
Model June-July-August mean climatology of the control experiment (SDE) for a) precipitation (mm day^−1^) and 850 hPa winds (ms^−1^), b) AOD, c) snow cover fraction, and d) seasonal cycle of land-sea contrast (°C), precipitation (mm day^−1^), AOD and snow mass, averaged over respective rectangular domain shown in a), b) and c) respectively. Land-sea contrast is computed as the temperature difference in the upper troposphere (500 −200hPa), between the northern [70–90°E, 20–30°N] and southern domain [70–90°E, 5°S-5°N]. Panels e), f), g), h) are the same as a), b), c) and d), except from observations, *i.e.* rainfall (TRMM), winds and temperature (MERRA2), AOD, and snow mass (MODIS) in normalized units.

To begin, we compare the model climatologies of precipitation, winds, aerosol optical depth (AOD), and snow cover over the ASM regions to observations. The model shows overall features representing a reasonably realistic monsoon climate system, with heavy monsoon precipitation over northern India, the Himalayan foothills and the western Ghats and prevailing low-level southwesterly winds over the region (Fig. 1a, e). High AOD over northern India and the Himalayan foothills is due mostly to dust transported from the deserts of the Middle East and West Asia, by the monsoon southwesterlies across the Northern Arabian Sea, and from the Thar deserts to the Himalayas foothills (HF), as well as local emissions from biomass burning and industrial sources over Indo-Gangetic Plain (Fig. 1b, f). Large snow cover fraction is found over the western Himalayas, southern and northern slopes of the Tibetan Plateau (Fig. 1c, g). Compared to observations, the model climatologies have notable discrepancies. Specifically, the model precipitation is excessive over the Himalayas foothills, but too weak, and not as well defined over the eastern Bay of Bengal/western Indo-China regions (Fig. 1a, e). These biases are most likely due to the inability of the coarse resolution of the GEOS-5 model to simulate orographic precipitation over complex terrains. Model AOD’s are too high over the Middle East, and northeastern India and Pakistan (Fig 1b, f). Snow cover is excessive over the western Himalayas, and northern slopes of the TP (Fig. 1c, d).

**Figure 2.**
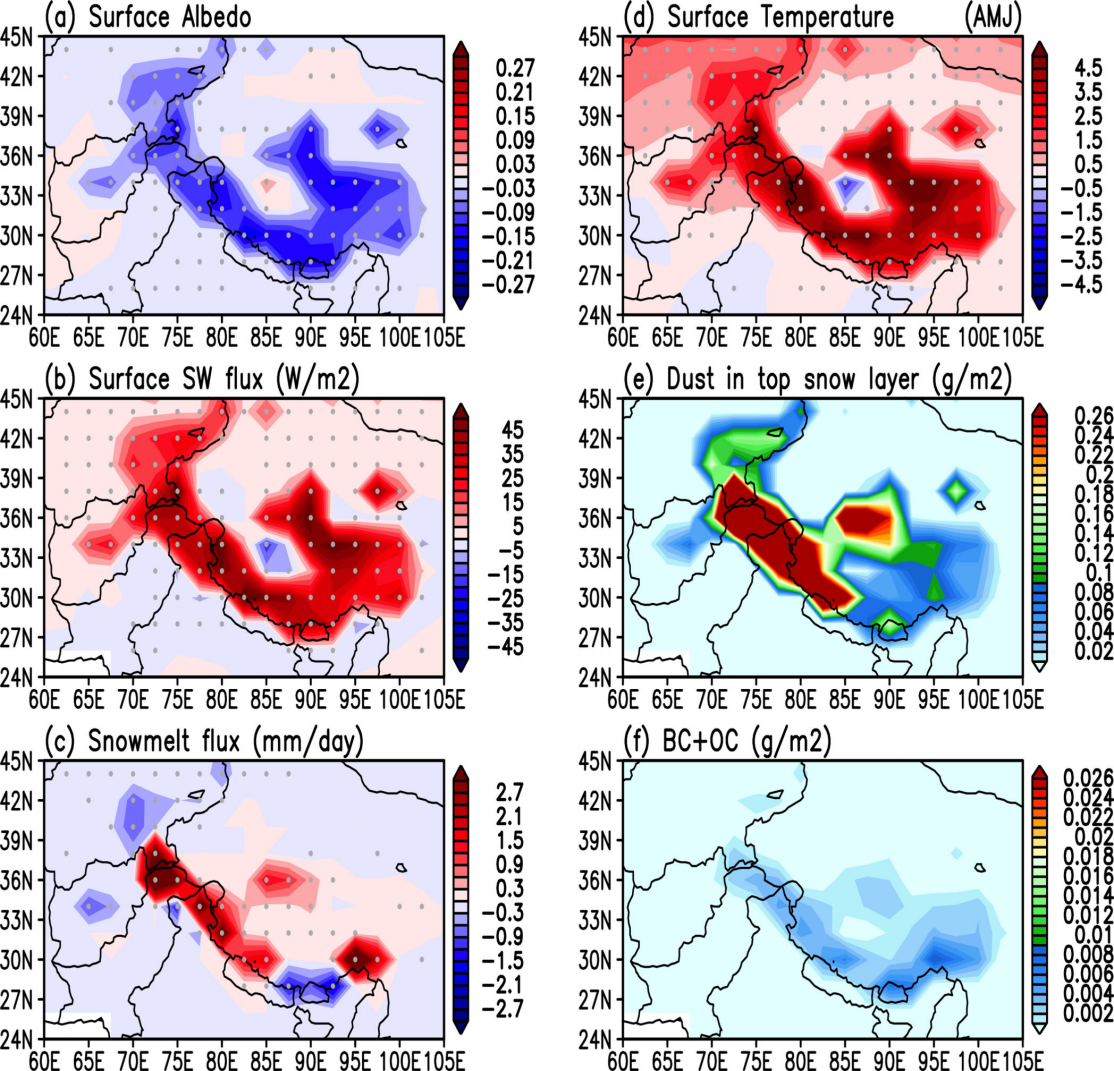
Anomalies induced by snow-darkening effects over the Himalayas-Tibetan Plateau region, for a) surface albedo, b) surface shortwave fluxes, c) snowmelt, d) surface temperature, e) dust in snow, and f) carbonaceous aerosols (OC +BC) in snow. Grey dots indicate 95% statistical confidence. Dust and carbonaceous aerosols in snow are set to zero in NSDE.

The climatological seasonal cycles of key monsoon control variables show good match between model and observation (Fig. 1d, h). Key features include increasing upper tropospheric meridional temperature gradient, and intensifying All-India rainfall (Fig. 1d, h, upper panels) during the late boreal spring and early summer monsoon (April-May-June), coincident with a rapid reduction in snow water equivalent (SWE), and increased AOD over northern Arabian Sea and northern India (Fig. 2d, h, lower panels). The model AOD peaks in April-May, in advance of the peak in rainfall (July), reflecting the seasonal progression of competing effects of aerosol emission, transport, and precipitation washout, in general agreement with ground-based observations from AERONET [72–74], but slightly in advance of MODIS AOD, which peaks in June-July. Also noteworthy is that the model has maximum snow cover in MAM, compared to JFM in MODIS observations. The reason for the temporal shifts in the model AOD and snow cover compared to MODIS are unclear. Worth noting here is that large uncertainties in snow cover and AOD in state-of-the-art global climate models, and in satellite retrievals still exist. Challenges in comparing model AOD and snow cover to satellite-derived estimates, and possible impact of model bias in our results will be discussed in the Conclusions (Section 4).

### 3.2 SDE induced forcing and feedback

**Figure 3.**
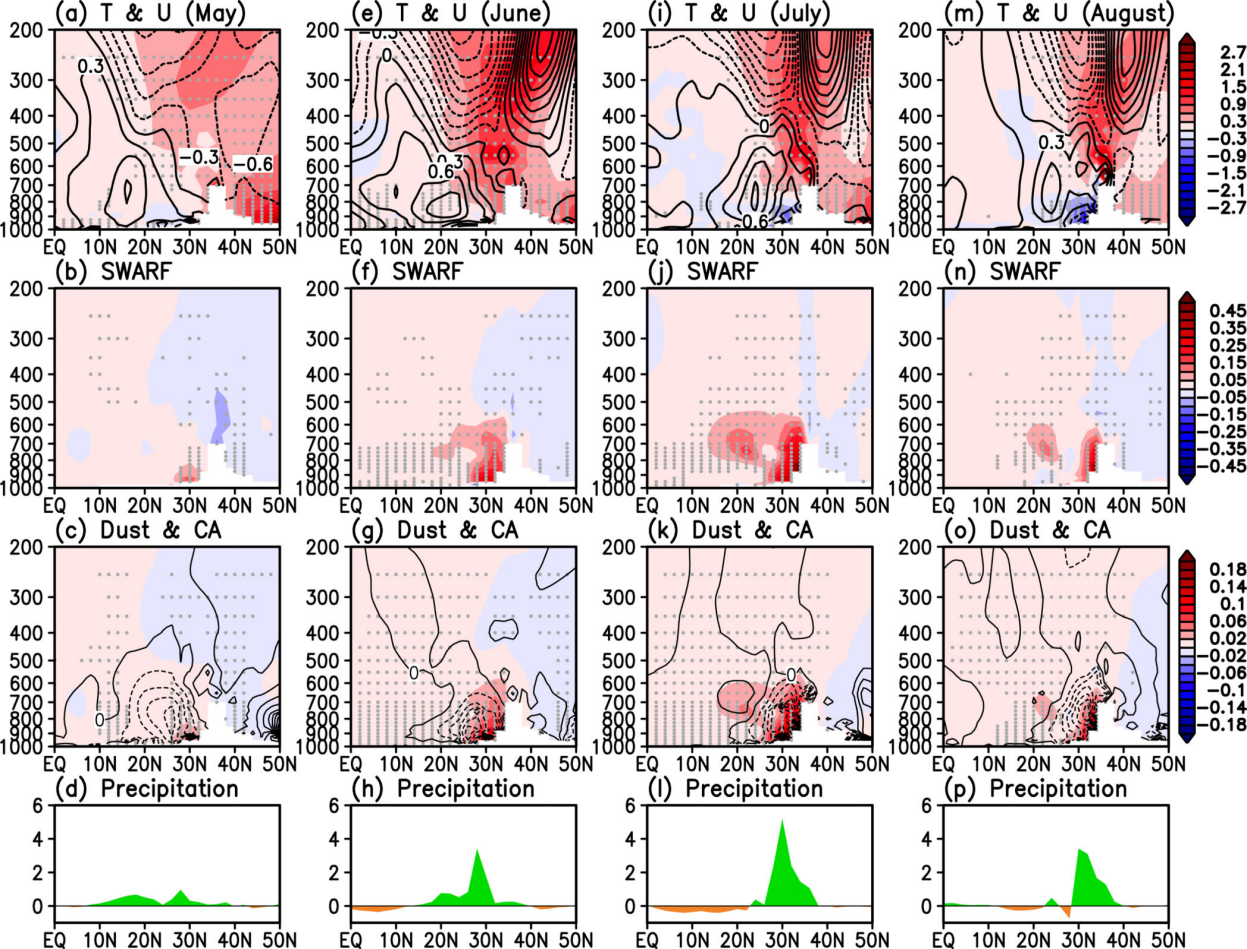
Height-latitude cross-section along 70–90 E, showing anomalies in a) temperature (° C, shaded) and zonal winds (ms^−1^, contoured), b) atmospheric heating by shortwave radiation (°C day^−1^), c) concentration of dust (color shaded in mg Kg^−1^) and CA (contoured with negative values dashed, in μg Kg^−1^ ), and d) precipitation (mm day^−1^) during May. Panels e), f), g) and h) are the same as a), b), c) and d), except for June. Same for i), j), k), l), and m), n), o), p), except for July, and August respectively. Grey dots indicate 95% statistical confidence level.

During the developing phase of the SASM in April-May-June (AMJ), SDE induces a strong reduction in surface albedo (up to 0.3), and increase shortwave (SW) absorption (+ 5–30 Wm^−2^) over the snow surface, most pronounced along the western, southern and eastern slopes of the HTP (Fig. 2a, b). The increased SW spurs rapid snowmelt and warming of the HTP land surface (Fig. 2 c, d). Also noteworthy is that in the southeastern and northwestern HTP regions, there are pockets of negative snowmelt flux (Fig. 2c), even though the SW surface forcing is positive. This is likely because of enhanced precipitation with enhanced convection and circulation, in the form of increased snowfall at higher elevations of the HTP during AMJ (see discussion pertaining to Fig. 3 in next subsection), that increases the snow amount, *i.e,* negative snowmelt flux, over some isolated regions where the rate of snowfall exceeds that of the snowmelt. As the snow cover is reduced, more areas of bare soil are exposed and the warming is accelerated through snow-albedo feedback [57]. As a result of SDE, the deposition of dust and CA in snow are also increased, especially over the Himalaya foothills (HF), which faces the increasing low-level monsoon southwesterlies (Fig. 2e, f, and Fig. 6b, later). Note that the large amplitude of the maximum surface warming (> 2°C), and related changes in accelerated snowmelt, loss of snow cover, as well as increased deposition of LAA’s in snow, are the result of full dynamical feedback of the coupled atmosphere-snow-aerosol system, which not only amplifies the initial local SDE warming over HTP surface, but also exerts influence over extended domains from surface to the upper troposphere spanning the greater ASM regions, lasting through entire monsoon season, as discussed next.

From May to June, the SDE-induced surface warming ramps up, extending to the upper troposphere over the HTP (Fig. 3a, e), by way of increased surface heat fluxes from the warmer land [75–76]. The temperature and wind changes are amplified by increasing shortwave aerosol radiative forcing (ARF) in the atmosphere abutting the HF (Fig. 3b, f), as well as by latent heating from increased precipitation over the region (Fig. 3d, h). The increased shortwave ARF stems from increased dust accumulation over the HF, as evident in their similar distributions indicating increased dust accumulation over the HF (Fig. 3c, g). The accumulation of dust in the HF is enhanced by remote transport by the increased low-level southwesterlies from the Middle East deserts, and at the same time, dust is removed by increased precipitation washout. The net increase in dust loading over the HF by SDE indicates that the accumulation out-weights the removal, resulting in a positive net ARF of the atmosphere. In contrast, CA, which are derived mostly from local emissions, are strongly removed due to SDE-induced increased precipitation washout, providing a negative heating feedback. Since the total ARF is positive in the HF region, the dust heating clearly out-weights any cooling effect due to the removal of CA. As a result of the aforementioned SDE forcing and feedback, the tropospheric warming peaks in June, in conjunction with the development of a north-south dipole anomaly in zonal winds above 400 hPa over the HTP, and strong anomalous westerlies (easterlies) below (above) 500 hPa over Indian subcontinent (10–30° N), signaling a strengthening SASM [77–81].

The warming of the upper troposphere, associated changes in winds, SW aerosol radiative heating, dust concentration and precipitation peak in July, over northern India, backing up against the southern slopes of the HTP (Fig. 3, i-l), and sustained through August (Fig. 3, m-p). Starting July, a cooling of the land surface and lower troposphere abutting the Himalaya foothills is noted. This is likely due to the blocking of surface radiation by increased transport of dust into the region, compounding by increased SW shielding by increased cloudiness and cooling by evaporation of falling rain in enhanced deep convection [82–84]. Most notable is that while the SDE is letting up due to the climatologically reduced snow cover in the HTP in July-August (See Fig. 1d, h), the anomalous shortwave aerosol radiative heating of the atmosphere in the HF remains strong, indicating that atmospheric heating by dust plays a major role in amplifying and strengthening the SASM during July-August. These features are consistent with the EHP mechanism for aerosol-monsoon dynamical feedback, which strengthening of the early SASM via atmospheric heating by LAAs [30, 34]. Here, we find additionally that SDE anchors the action center of the EHP to the southern HTP and HF where the SDE effect is most pronounced, intensifying the monsoon not only during the early monsoon, but through the entire monsoon season. Also noteworthy is that while these results are robust in the GEOS5 model, the model has excessive bias in HTP snowcover and AOD, compared to observations. More discussion on how the biases may affect the model results are presented in the conclusions (Section 4).

### 3.3 Changes in mean monsoon climate

**Figure 4.**
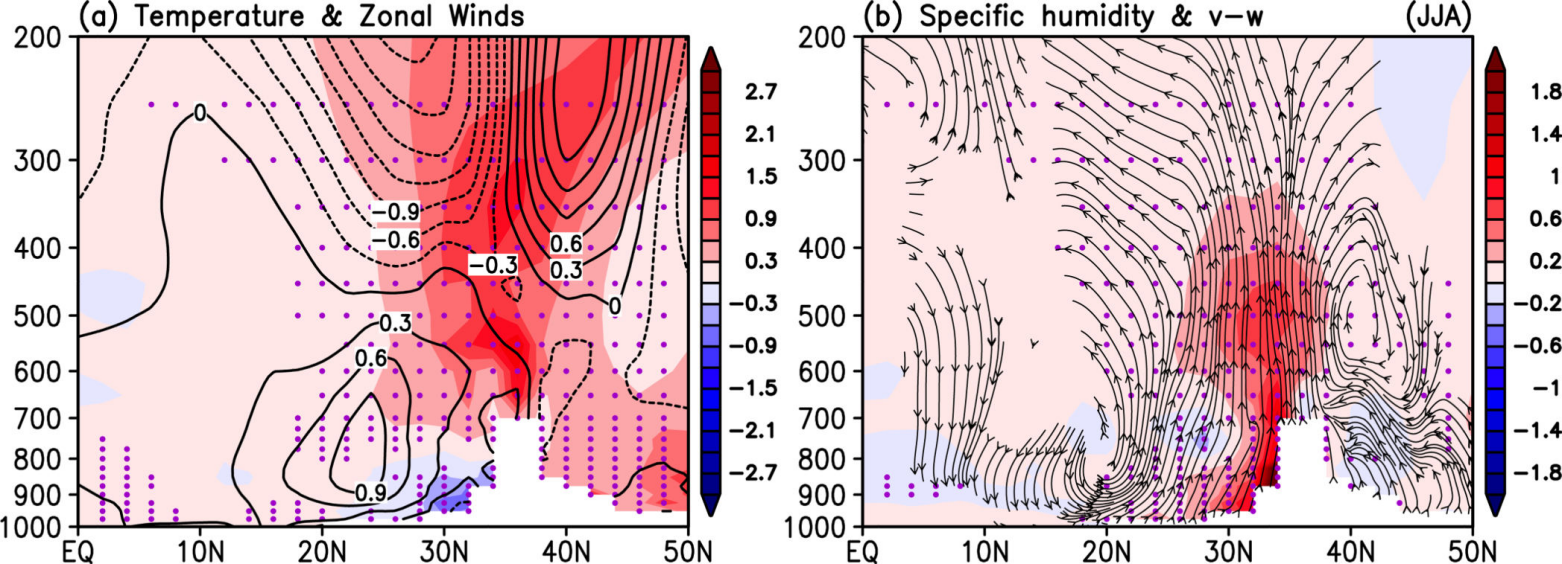
Height-latitude cross-section June-July-August mean anomalies, averaged over (70–90° E) for a) temperature (° C) and zonal winds (ms^−1^), and b) specific humidity (gKg^−1^), and streamlines of anomalous meridional circulation. Purple dots indicate 95% statistical significance.

As a result of the combined effects of SDE SW forcing and dynamical feedback, the seasonal mean SASM is strengthened as indicated by a well-developed warm anomaly in the upper troposphere over the HTP, sandwiched between a dipole zonal wind anomaly, with increasing easterlies in the tropics 10–30° N, and increasing westerlies in the extratropics 35–50° N (Fig. 4a), signaling a strengthening of the Tibetan Anticyclone (TPA) [77–78, 80–81]. Another evidence of a strengthened

SASM can be found in the increased vertical easterly shear with enhanced low-level westerlies in the lower troposphere below 400 hPa and increased upper level easterlies above at 15N −30° N [79, 85]. In conjunction with the changes in zonal winds and temperature, an enhanced monsoon meridional circulation, featuring increased moistening of the lower and mid- troposphere by anomalous low-level southerlies and deep rising motions over northern India and the HTP. The ascending moister air is coupled to anomalous sinking of drier air over southern India and the northern Indian Ocean (0–15°N), and north of the HTP (45–50° N).

**Figure 5.**
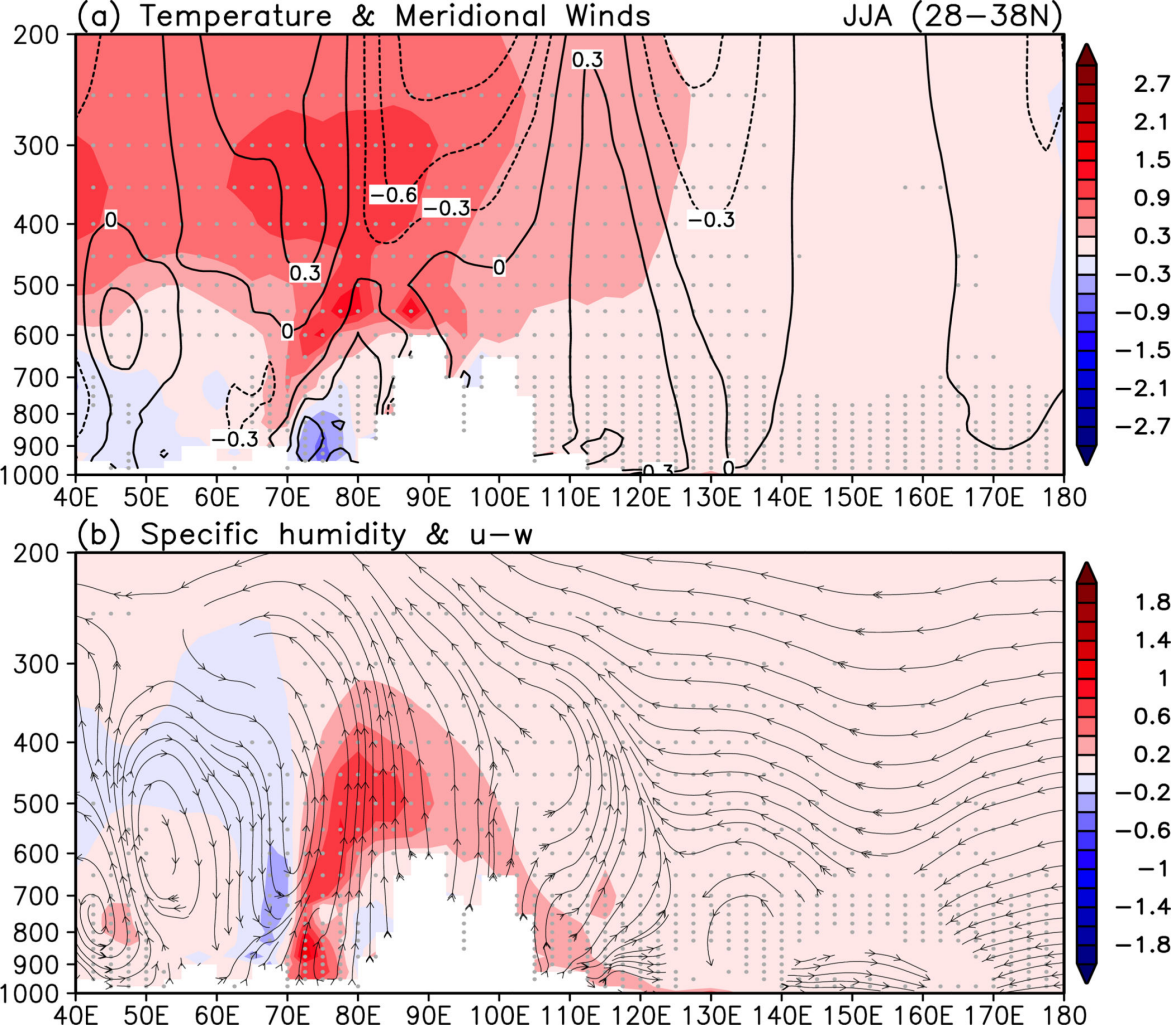
Height-longitude cross-section of June-July-August mean anomalies averaged over (28–38° N) of a) temperature (°C) and meridional winds (ms^−1^), and b) specific humidity (gKg^−1^) and streamlines of anomalous east-west circulation spanning the Middle East to the western Pacific (40° E – 180°). Statistical significant exceeding 95% are indicted by grey dots.

**Figure 6.**
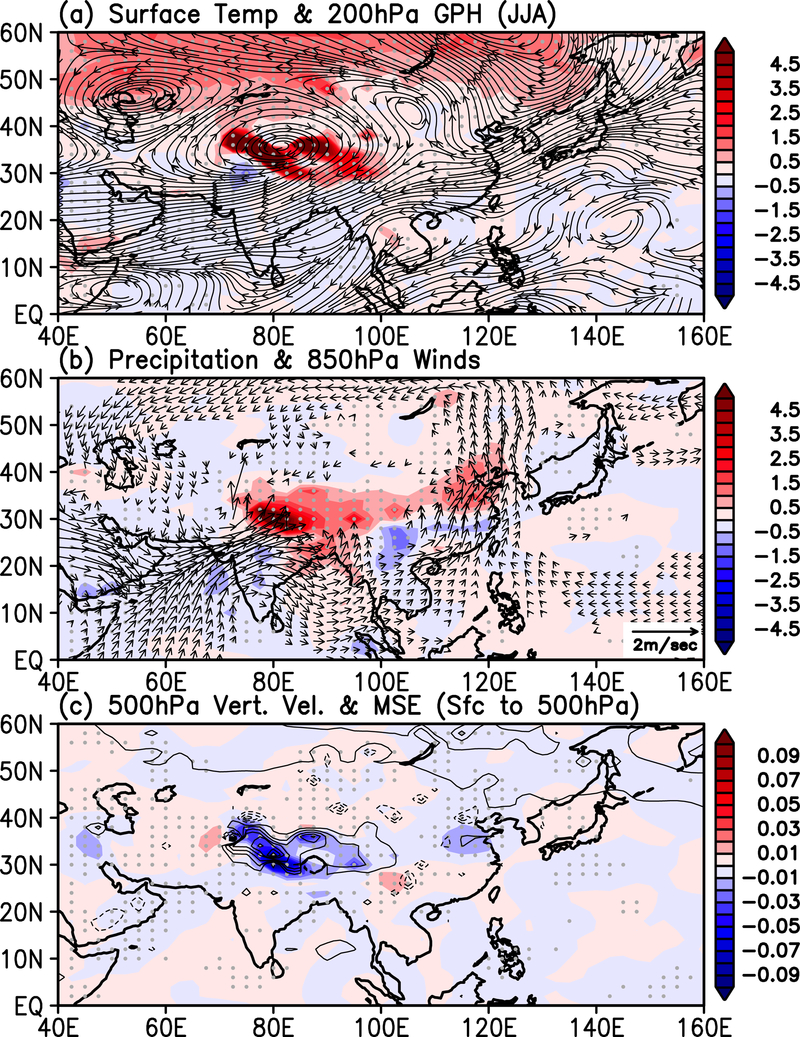
Horizontal distributing showing anomalies in a) surface temperature (°C), and geopotential height at 200hPa, b) precipitation (mmday^−1^) and 850 hPa winds (ms^−1^), and c) vertical motion at 500 hPa (color-shading, in units of Pa s^−1^) and lower troposphere moist static energy (contours, in units of watt m^−2^)

The SDE in the HTP region impacts not only the SASM, but also the greater ASM regions. The warming of the mid- and upper troposphere arising from the center of action over the high-terrain HTP region (70–90°E) is expansive (Fig. 5a), spanning the entire Middle East and Asian monsoon domains (40– 120° E). Here, the maximum warming straddles enhanced northerlies (southerlies) to the west (east), consistent with an enhancement of the TPA over the HTP. Emanating from the enhanced TBA is a pattern with alternating meridional winds with opposite signs in the mid- and upper troposphere, spanning a wide range of longitudes (40–130°E). The meridional wind pattern is associated with alternating deep tropospheric rising and sinking motions from the Middle East across South Asia to East Asia (Fig. 5b). Increased atmospheric moisture and anomalous rising motions are found most pronounced over the western HTP in northern India/Pakistan (70–90° E), and to a lesser extent, on the eastern slopes of the HTP and central central-northern East Asia (100–120° E). Further details on the distribution of vertical motions will be discussed with reference to Fig. 6, later. Over the East Asia, the east-west circulation exhibits a strong westward tilt with height, reflecting the baroclinic tendency of mid- to upper tropospheric extratropical westerlies and interactions with low-level moisture transport, and moisture convergence on the *Mei-Yu* front of EASM [85–88].

The aforementioned temperature and circulation features are associated with the formation of an anomalous Rossby wavetrain, spanning northern Eurasian (30–60°N, 40–140°E), with an enhanced anomalous TPA anchored to the surface heating of the HTP, connected to alternating cyclonic and anticyclonic circulation cells, over northern China/Mongolia, and northeastern East Asia respectively (Fig. 6a). The Rossby wavetrain occurs in conjunction with an elongated band of anomalous easterlies stretching from the Sea of Okhotsk, across Japan and central China, signaling a weakening of the climatological subtropical East Asian jet (120–160E). The anomalous easterlies continue westward, merging with the southern flank of the enhanced TPA, and further on across the Middle East. The planetary scale nature of the easterly wind anomalies is associated with a warming of the northern Eurasia continent in boreal spring and early summer [56–57], which pre-conditions the snow cover change and warming over the HTP during JJA (Fig. 6a). As a result of SDE induced warming over the HTP, the rainfall and circulation patterns of the entire ASM are substantially altered. Increased low-level westerlies transport more dust aerosols from the desert regions across the North Arabian Sea into India, accumulating them over the HF, enhancing the SASM through the EHP positive feedback mechanism. As a result of increased moisture transport by the southwesterlies, precipitation is strongly enhanced over the HTP and northeastern India. The latent heat release from increased precipitation further enhances the warming of atmosphere over the HTP. Over East Asia, precipitation is increased over northern and northeastern China due to the increased southerly moisture transport from the south, from Indo-China and the South China Sea. For both the SASM and the EASM, a dipole anomalous precipitation pattern (north-positive and south-negative) is found, signaling an intensification, and northward displacement of the climatological monsoon rain belt. Over East Asia, this displacement may signal the “abrupt northward jump” of the *Mei-Yu* rainbelt from central to northern China [86–88}. The main driver of the increased rainfall stems from the increased low-level moist static energy (MSE), with a pronounced primary action center over the southern slopes of the HTP, and a secondary center over northeastern China (Fig. 6c). These centers feature strong anomalous mid-tropospheric ascent, due to increased latent heating, and orographic uplifting on wind facing steep slopes of the HF regions (Fig. 6c), as well as strong low-level transport of moisture from the Arabian Sea, and from Southeast Asia/South China Sea respectively. Because of the stable air near the tropopause, the increased ascent over the HTP leads to the shrinking of the air column above, and the development of the anomalous anticyclonic center, enhancing the TPA [26, 76].

### 3.4 A possible mechanism for the Blanford Hypothesis

**Figure 7.**
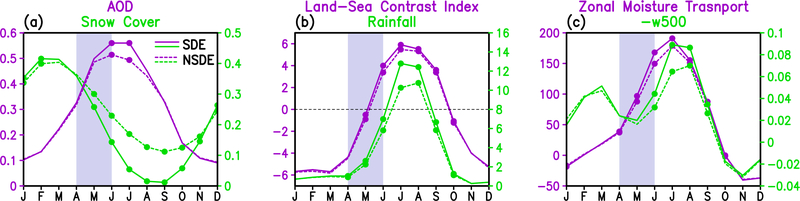
Time series depicting the seasonal cycles of a) snow cover (percentage) and AOD, b) land-sea contrast (°C) and rainfall (mm day^−1^) over northern India (70–90°E, 25–35°N), and c) westerly low-level (1000–850hPa) moisture flux (Kg m^−1^s^−1^) averaged over the eastern Arabian Sea (70–75° E, 10–30° N) and negative p-velocity (Pa s^−1^) over the northern India. Domains for snow cover and AOD are the same as used in Fig. 1. Labels and ordinate units are color-matched to the line plot.

The physical processes underlying changes in the seasonal cycles of key elements of the aerosol-snow-monsoon climate system induced by SDE over the HTP for the SASM are summarized in the context of the Blanford hypothesis (Fig. 7). During April-May-June, increased snowmelt and reduction in snow cover over HTP are induced by deposition of LAAs on snow cover surface. The impurities in snow reduces surface albedo and enhances the absorption of insolation, leading to rapid snowmelt, loss of snow cover and warming of the land surface, and the atmosphere over the HTP (Fig. 7b). As the monsoon season advances in May-June, the warming over the HTP is amplified by aerosol-radiation-circulation-precipitation feedback, rapidly extending throughout the upper troposphere over the HTP, increasing the meridional temperature gradient between the monsoon and oceanic regions to the south (Fig. 7b), resulting in increased precipitation over northern India in the early monsoon season [89–91]. The increased southwesterlies associated with the strengthened early monsoon enhances the dust loading and atmospheric heating over the Himalaya foothills region in northern India, sustaining the feedback through the aerosol “Elevated Heat Pump” mechanism [30–31] through July-August, even when the climatological snow cover over the HTP is significantly reduced (Fig. 7a). As a result of the SDE induced dynamical feedback, the monsoon rainy season is advanced, and the monsoon precipitation over northern Indian/HTP region is strongly enhanced in July-August (Fig. 7b). Correspndingly, the moisture flux from the East Arabian Sea into the India subcontinent increases in AMJ, peaking in July, while the ascending motion at 500hPa over the northern India/HTP region substantially enhanced in JJA (Fig. 7c), in unison with the precipitation increase there (Fig. 7b). Interestingly, the climatological vertical motion over the region show steady increase from November-March and then a decline in March-May (Fig. 7c). A close examination of the seasonal variation of the winds indicates that the seaonal varation in vertical motion over this region is most likely due to orographic forcing of the mid-to-upper level westerlies by the TP, which are strongest in boreal winter. As the monsoon season approaches, the upper westerlies migrate poleward to north of the TP, and the vertical motion weakens. However, starting in May, heating over the TP, and increased moisture transport by the low-level monsoon southwesterlies lead to increased ascent over northern India/HTP. There is no different between SDE and NSDE. Indeed, all aforementioned key monsoon indicators signal a strengthening SASM from May through August, due to SDE-induced reduction in snowcover, but with minimal or no impacts over these regions during the rest of the year. Given the time lagged relationship between snow cover and increased monsoon rainfall, it is possible that such a relationship could have potential value for prediction of the strength SASM, as first proposed by Blanford more than a century ago. Based on these relationships, it can be argued that monitoring dust conditions over the Arabian Sea, and Northern India during the pre-monsoon season (April to mid-June) could provide value-added information for seasonal-to-interannual predictability of the SASM.

## 4. Conclusions

Based on numerical simulations using the NASA GEOS5 climate model, we have examined the possible impact of snow-darkening effects (SDE) by deposition of light absorbing aerosols (LAAs) on snow cover over the Himalayan-Tibetan Plateau (HTP), and subsequent influence on the Asian summer monsoon. Results show that during April-May-June, LAA’s deposition on snow reduces surface albedo, increases absorption of surface shortwave radiation, reduces snow cover by rapid snowmelt, and induces a strong surface warming (> 2° C) in the western, southern and eastern flanks of the HTP. The surface warming extends from the HTP surface to 200 hPa and above, through dynamical feedback processes, in associated with an enhanced Asian monsoon, featuring a stronger Tibetan Plateau Anticyclone (TPA), with increased low-level southwesterly flow and increased precipitation over northern India. Increased dust aerosols are transported from the Middle East, and West Asia and Thar deserts by the strengthened monsoon southwesterlies to the Himalayan foothills (HF) and the Indo-Gangetic Plain (IGP) of northern India. The increased dust transport from remote sources overpowers the wet removal of dust by increased precipitation, resulting a net increase in dust loading over the IGP. On the other hand carbonaceous aerosols, which are derived mostly from local sources in the IGP and HF, are strongly removed by increased precipitation washout. The net accumulation of dust aerosols in the IGP and HF plays an important role in heating of the atmosphere by shortwave radiative forcing, which is reinforced and sustained by increased latent heating from enhanced precipitation over northern India through July-August, via the Elevated Heat Pump (EHP) mechanism [30–31].

The SDE-induced dynamical feedback leads to a new equilibrium monsoon climate. During JJA, the strong warming over the HTP, excites an upper tropospheric wavetrain, with alternating cyclonic and anticyclonic circulation cells that span eastern Europe and East Asia. Anomalous circulation cells develop along the wavetrain is responsible for a weakening of East Asian jet, and enhancement of the TPA, that is coupled to increased poleward transport of moisture that spurs a northward shift, and intensification of precipitation over India and East Asia. We find that the SDE induced rapid snowmelt and warming over the HTP can effectively anchored the EHP dynamical feedback, via strong build-up of moist static energy, anomalous ascent and latent heating in the southern slopes of the HTP, where orographic forcing by the steep topography can provide efficient penetrative convection transporting heat, moisture and aerosols to the upper troposphere and lower stratosphere [92].

As a caveat, we note that simulations of monsoon mean states of meteorology, snow cover and aerosol, and comparison with observations by state-of-the-art climate models, including the GEOS5 model used in this study are still challenging, due to inadequate model physics, as well as large uncertainties in aerosol and snow products from satellite retrievals [93–96]. In the present study, for computation economy of long-term (100 years) simulation, we used a low-resolution version of the GEOS5 model, and emphasized the large-scale interactions. While the model possesses a reasonable monsoon climate, it has non-negligible discrepancies when compared with observations, in that the AOD is too high, snow cover is too much, rainfall is over-estimated over the HF and underestimated in the eastern Bay of Bengal. The GEOS5 model used here is also known to have cold bias over continents [56–57], due to excessive snow cover in Eurasia and the HTP. In the real world, the SDE direct (radiative) forcing is time and space limited. Once the ground snow is all melted, the SDE shortwave radiative forcing vanishes, even though the induced anomalies may continue to be amplified by dynamical feedback. In the model, the excessive snow cover means that SDE continues to have an effect even during the peak monsoon season. Hence the excessive model AOD and snow cover in boreal spring through summer may mean an over-estimation of the SDE effects on monsoon compared to the real world, especially when other control factors such as anomalous sea surface temperatures forcing are in play.

Nonetheless, from the idealized experiments, our results have shed new light on a possible physical mechanism that underpins the Blanford hypothesis, *i.e.,* increased (decreased) deposition of LAAs on HTP snow surface can lead to a decrease (increase) in surface albedo resulting in in less (more) snow cover, warming (cooling) of the HTP land and atmosphere, enhanced (reduced) westerly transport of desert dust from the Middle East desert across the North Arabian Sea, and increased (decreased) dust loading and heating of the atmosphere over the HF and the IGP in April-May-June, foreshadowing a robust increase (decrease) in rainfall over northern India in July-August. Given the availability of much improved and reliable multiple data sources from in-situ, satellite and reanalysis, specific information on snow cover and aerosols could be systematically diagnosed and incorporated into empirical and model monsoon forecasts, as first envisioned by Blanford (snow cover only) over a century ago. Snow cover and related conditions (temperature, soil wetness and others) over Eurasia in spring and early summer could also be implemented to improve monsoon seasonal-to-interannual forecasts.

Finally, the results of this study support the new paradigm that LAA’s such as desert dust and BC and OC from biomass burning which are abundant in monsoon regions from natural sources, are intrinsic components of a monsoon climate system, contributing significantly to the distribution heat sources and sinks of the monsoon on multiple time scales [41, 97]. Better understanding of interaction of monsoon dynamics and ambient aerosols (natural and anthropogenic) are essential in further unraveling the causes, consequence and predictability of climate variability and change of in monsoon regions.
